# Regenerative Potential of Mesenchymal Stem Cells for Enhancing Uterine Health and Fertility in Repeat Breeder Dairy Cows

**DOI:** 10.1155/sci/8863818

**Published:** 2026-02-06

**Authors:** Bruno Leonardo Mendonça Ribeiro, Joice Fülber, Mario Augusto Reyes Aleman, Luiz Francisco Machado Pfeifer, Jéssica de Souza Andrade, Elizângela Mírian Moreira, Renata Reis de Silva, Raquel Yvonne Arantes Baccarin, Lilian Rose Marques de Sá, Jade Li, Lilian Gregory

**Affiliations:** ^1^ School of Veterinary Medicine, Federal University of Mato Grosso (UFMT), Sinop Campus, Mato Grosso, Brazil, ufmt.br; ^2^ Department of Internal Medicine School of Veterinary Medicine and Animal Science of University of São Paulo, University of São Paulo (USP), São Paulo, Brazil, usp.br; ^3^ Brazilian Agricultural Research Corporation (EMBRAPA), Porto Velho, Rondônia, Brazil, embrapa.br; ^4^ Postgraduate Program in Biodiversity and Biotechnology, Bionorte, Porto Velho, Rondonia, Brazil; ^5^ Federal University of Amazonas (UFAM), Humaitá, Amazonas, Brazil, ufam.edu.br

**Keywords:** bovine, histopathology, infertility, repeat breeding, stem cell therapy

## Abstract

The suboptimal reproductive performance of repeat‐breeding (RB) cows is a major challenge for the dairy industry, leading to higher costs, prolonged calving intervals, and reduced productivity, negatively impacting herd productivity and economic viability. Among the associated factors, endometrial degeneration stands out, characterized by the replacement of functional tissue with fibrotic tissue, compromising uterine receptivity. In this context, regenerative therapy using mesenchymal stem cells (MSCs) has emerged as a promising alternative. This study evaluated the effects of intrauterine MSC inoculation in RB cows diagnosed with endometrial degeneration. Nine crossbred cows (Gyr × Holstein) were included and underwent clinical, cytological, microbiological, histopathological, ultrasonographic, and molecular evaluations on day 0 (pre‐treatment) and day 30 (post‐treatment). The results demonstrated endometrial remodeling, with fibrotic tissue replaced by loose connective tissue, increased vascularization, and the presence of new groups of endometrial glands. Doppler ultrasonography revealed enhanced blood flow of the endometrial mucosa and thickening of the uterine wall after therapy. qRT‐PCR analysis indicated reduced expression of pro‐inflammatory cytokines (IL‐1*β* and IL‐8), suggesting modulation of the uterine environment. Despite the observed tissue improvement and absence of adverse effects on ovarian function, none of the inseminated cows conceived. In conclusion, MSC therapy promoted favorable changes in the endometrium and uterine environment, although it did not result in pregnancy, highlighting the need for further studies to optimize dosage, administration route, and therapeutic response time.

## 1. Introduction

Dairy farming is a major economic activity, yet reproductive performance issues can significantly impact productivity and profitability [1, 2]. Reproductive inefficiency in repeat‐breeding (RB) cows, characterized by regular estrous cycles without conception, represents a costly challenge for farmers by prolonging breeding intervals, extending calving periods, and ultimately reducing both calf birth rates and milk production [3–5].

RB is one of the most prevalent reproductive disorders in dairy cattle, defined as the failure to conceive after three or more artificial or natural inseminations, often without an identifiable cause [6, 7]. However, RB has been associated with various factors, including reproductive tract infections, advanced maternal age, anatomical abnormalities, nutritional deficiencies, hormonal imbalances, estrus detection failures, inadequate uterine receptivity, and disruptions in maternal–conceptus interaction and implantation processes [7–16]. Additionally, previous studies have shown that subclinical conditions, such as inflammation, may compromise uterine health and negatively impact pregnancy rates, particularly when polymorphonuclear (PMN) cell counts and proinflammatory cytokine expression levels are elevated [17–19].

Among these factors, endometrial degeneration, characterized by the replacement of functional tissue with fibrous tissue, has received increasing attention in the literature, particularly due to its direct association with failures in embryo implantation and pregnancy maintenance [20, 21]. In many cases, morphological alterations of the endometrium can only be detected through histopathological examination [22], making its diagnosis a challenge in clinical practice [23]. Given the progressive and often irreversible nature of these lesions, conventional therapeutic approaches [24–27] show limited efficacy, highlighting the need for the development of more effective and regenerative strategies.

In this context, regenerative medicine, through the application of mesenchymal stem cells (MSCs), emerges as a promising alternative. MSCs are multipotent cells capable of differentiating into various cell types and performing immunomodulatory, anti‐inflammatory, and angiogenic functions. Additionally, they are known to secrete trophic factors that promote tissue repair, cellular regeneration, and extracellular matrix remodeling, possessing great potential to repair and regenerate aged or damaged tissues [28].

Research has identified stem cells within the human myometrium, contributing to uterine remodeling, particularly during pregnancy [29, 30]. In veterinary medicine, studies involving MSCs in reproductive contexts have intensified in recent years, particularly in equine species, where cell therapy has already demonstrated the ability to reverse chronic degenerative endometritis, stimulate glandular epithelial proliferation, and improve uterine vascularization [31]. In ruminants, however, the use of MSCs remains incipient, with few studies exploring their practical application in endometrial regeneration in cows suffering from infertility associated with uterine fibrosis. Although initial results are promising, significant challenges still persist, including the standardization of dosage, routes of administration, therapeutic response time, and objective evaluation of both histological and functional outcomes.

Ongoing research in this field highlights the growing relevance of stem cell therapy as a transformative approach to the treatment of reproductive disorders in dairy cattle, with potential benefits for both the dairy industry and animal welfare.

Given this context, it is pertinent to investigate the potential of MSC therapy in the treatment of infertility in RB cows, particularly in those diagnosed with endometrial degeneration. The ability of MSCs to modulate the uterine environment, reduce inflammation, and stimulate tissue regeneration may represent a viable alternative for restoring endometrial function and improving reproductive performance.

Therefore, the present study aimed to evaluate the effects of intrauterine inoculation of MSCs in RB cows diagnosed with endometrial degeneration, with the goal of contributing scientific evidence regarding the applicability of cell therapy in the reproductive management of dairy cattle, focusing exclusively on its implications for bovine reproduction and productivity, without the inclusion of humane endpoints.

## 2. Materials and Methods

The work has been reported in line with the ARRIVE guidelines 2.0. This work was approved by the Ethics Committee on Animal Use of the School of Veterinary Medicine and Animal Science (University of São Paulo; CEUA/FMVZ) with Protocol Number CEUA 2489230217.

### 2.1. Animals and Study Design

This study was conducted at the experimental research farm of Embrapa Rondônia (Brazilian Agricultural Research Corporation, Rondônia, Brazil; 08°48′12″ S, 63°50′56″ W). A total of 10 nonlactating crossbred dairy cows (Gyr × Holstein), aged between 3 and 6 years, with a body condition score (BCS) of 3–4 on a 5‐point scale (where 1 = emaciated and 5 = obese; Lowman et al., [32], were used. The cows were maintained in an outdoor grazing system on *Brachiaria brizantha* pasture with ad libitum access to mineral salt and water.

All cows included in this study had undergone more than five timed artificial insemination (TAI) protocols without achieving pregnancy and were classified as repeat breeders. Selection criteria were based on their history of reproductive failure, microbiological and cytological analyses, and histopathological confirmation of endometrial degeneration. These evaluations were performed 30 days before MSC treatment (Day 0; D0; untreated). The cows then received an intrauterine infusion of MSCs, and a second uterine tissue evaluation was conducted 30 days posttreatment (Day 30; D30; treated). A separate control group was not established; instead, each cow served as its own baseline, with pretreatment evaluations serving as the comparative reference.

### 2.2. Collection and Cultivation of MSCs

MSC collection and cultivation were performed by harvesting ~15 mL of bone marrow from a healthy bovine donor. For bone marrow collection, the donor animal was sedated intravenously with xylazine (Rompun, São Paulo, Brazil) at a dose of 0.02 mg/kg. A trichotomy was performed in the lumbosacral region to access the iliac crest. Following the identification of the collection site, local anesthesia was administered using lidocaine (Lidovet, Rio de Janeiro, Brazil) at a dose of 5 mg/kg without a vasoconstrictor in the subcutaneous tissue. The site was then aseptically prepared with 2% chlorhexidine scrub (Riohex 2%, São José do Rio Preto, Brazil) followed by alcoholic chlorhexidine (Riohex 0.5%, São José do Rio Preto, Brazil).

**Figure 1:**
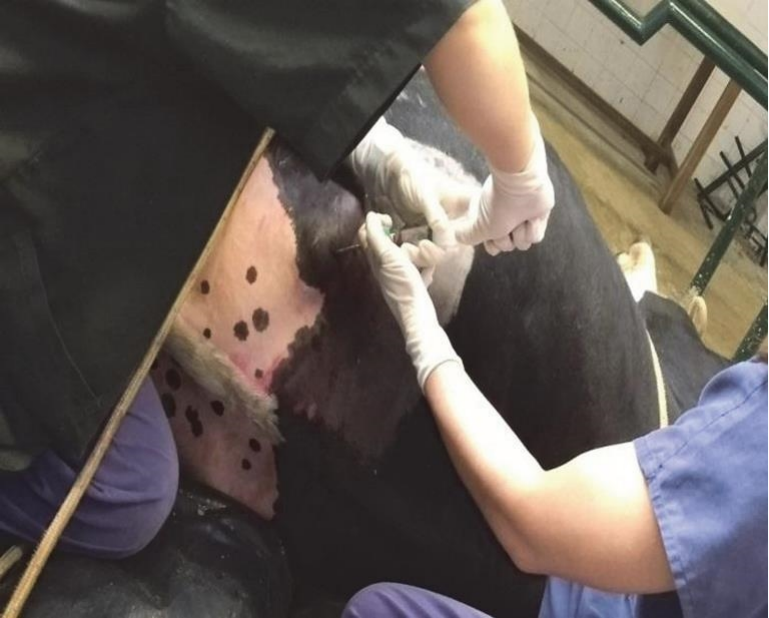
Mesenchymal stem cell collection, intramedullary lumbosacral region.

Bone marrow aspiration was performed using an 8‐gauge, 15 cm Komiyashiki needle inserted dorsoventrally, perpendicular to the skin, as shown in Figure 1. Once the needle was in place, ~15 mL of bone marrow was aspirated using a 20 mL syringe preloaded with 1 mL of heparin (Hemofol, Itapira, Brazil) to prevent coagulation. The collected samples were immediately stored in a Styrofoam container with crushed ice for preservation until further laboratory processing. MSCs were subsequently isolated, expanded, enzymatically dissociated, and cryopreserved in liquid nitrogen at the third passage (P3).

The cell extraction procedure involved diluting bone marrow in phosphate‐buffered saline (PBS; Ambion, Waltham, MA, USA) at a 1:1 ratio and gently layering it over Ficoll Histopaque solution (density 1.077 g/mL; Sigma–Aldrich, St. Louis, MO, USA, Catalog No. 10771) in equal proportions. The mixture was then subjected to centrifugation at 400 × g for 30 min at 24°C to separate cells based on their density. The mononuclear cell fraction was carefully resuspended in PBS and centrifuged at 720 × g for 10 min, followed by a second centrifugation at the same speed for 10 min. The resulting pellet was transferred to 25 cm^2^ culture flasks and cultured in DMEM/F12 medium (Gibco, Waltham, MA, USA), supplemented with 10% fetal bovine serum (Gibco), 1% penicillin (10,000 U/mL; Agrosil PPU, Descalvado, Brazil), 10 mg/mL streptomycin (Estreptomicina Biofarm, Jaboticabal, Brazil), 25 µg/mL amphotericin B (Anforicin B, Itapira, Brazil), and 200 mM glutamine (Gibco). The cells were incubated at 37°C in a humidified atmosphere (100% relative humidity) with 5% CO_2_. The culture medium was replaced every 48 h until 70%–80% confluence, as recommended by Smith et al. [33].

Following the production and storage of MSCs, they were transported over a 24 h period in a dry shipper (Thermo), which maintained a temperature below −150°C, essential for preserving cell integrity and halting metabolic activity.

The isolated cells exhibited plastic adherence and a fibroblast‐like morphology, which are hallmark characteristics of MSCs and can be seen in Figure 2. Furthermore, multipotency was confirmed through differentiation assays, demonstrating their capacity to differentiate in vitro into three mesodermal lineages: adipocytes, osteocytes, and chondrocytes, as shown in Figure 3. The phenotypic characterization of the cells was performed by flow cytometry using a FACSCalibur cytometer (Becton Dickinson, San Jose, CA, USA) operated with the CellQuest software (Becton Dickinson, San Jose, CA, USA). The cell samples were incubated with fluorochrome‐conjugated monoclonal antibodies, as specified below: mouse antihuman CD90–FITC, mouse antirat CD73–PE, rat antimouse CD105–PE, mouse antidog CD34–FITC, mouse antisheep CD45–FITC, and mouse antisheep MHC‐II–FITC. To control for nonspecific fluorescence and to adjust compensation parameters, the isotypes anti‐IgG1–PE and anti‐IgG1–FITC were employed. All labeling and acquisition procedures strictly followed the manufacturers’ recommendations. The selected antibody set was organized to form an immunophenotypic panel aimed at identifying and confirming the typical characteristics of MSCs.

Figure 2Representative images of bovine mesenchymal stem cell cultures showing cells adhered to plastic and displaying a fibroblast‐like morphology. (A) 10× magnification; (B) 20× magnification.

**Figure:**
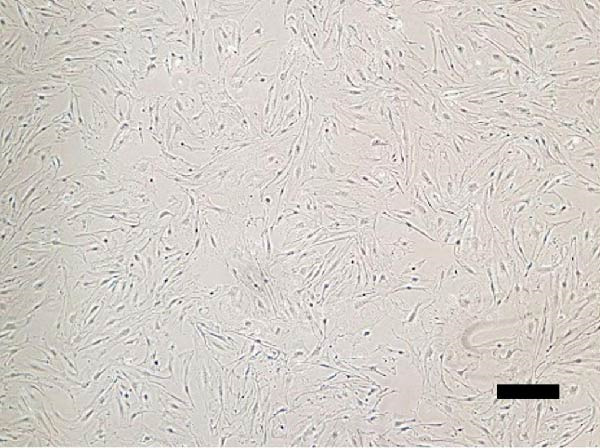
(A)

**Figure:**
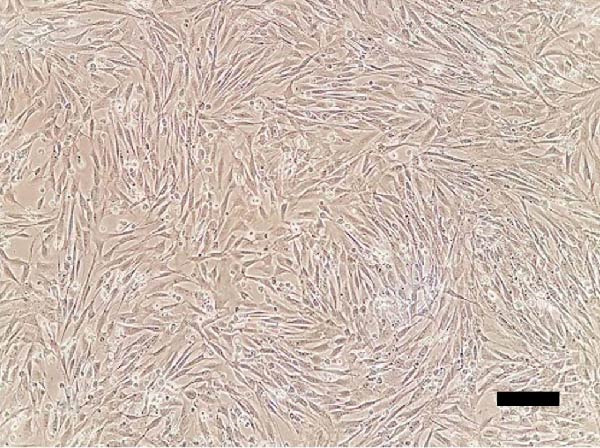
(B)

Figure 3Representative images of bovine mesenchymal stem cell differentiation. (A) Osteogenic differentiation, 10× magnification; (B) negative control for osteogenic differentiation, 10× magnification; (C) adipogenic differentiation, 20× magnification; (D) negative control for adipogenic differentiation, 20× magnification; (E) mesenchymal stem cells with chondrogenic differentiation, 20× magnification; (F) mesenchymal stem cells with chondrogenic differentiation, 10× magnification.

**Figure:**
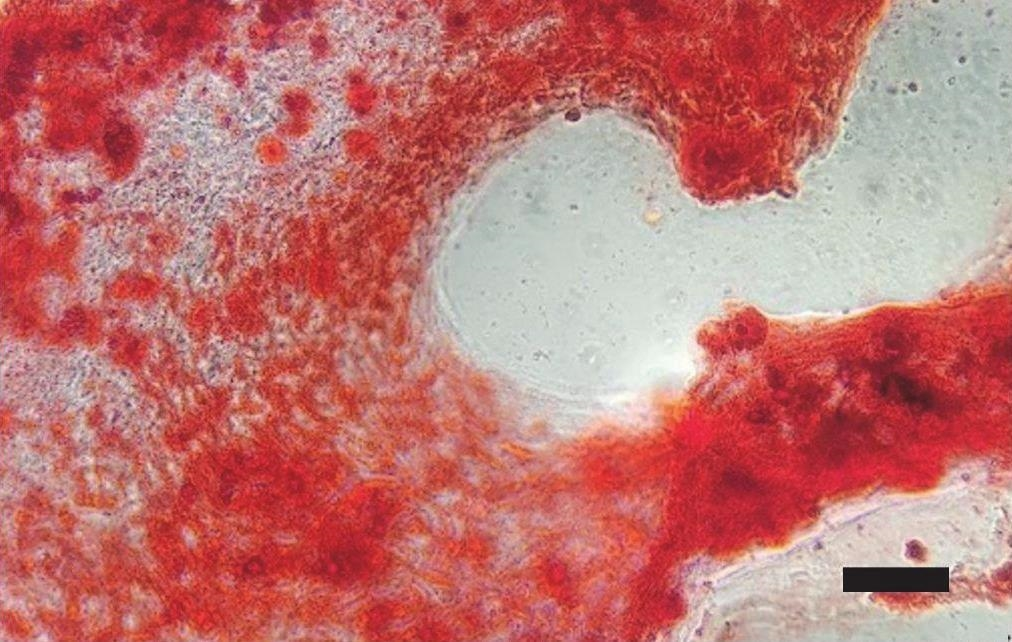
(A)

**Figure:**
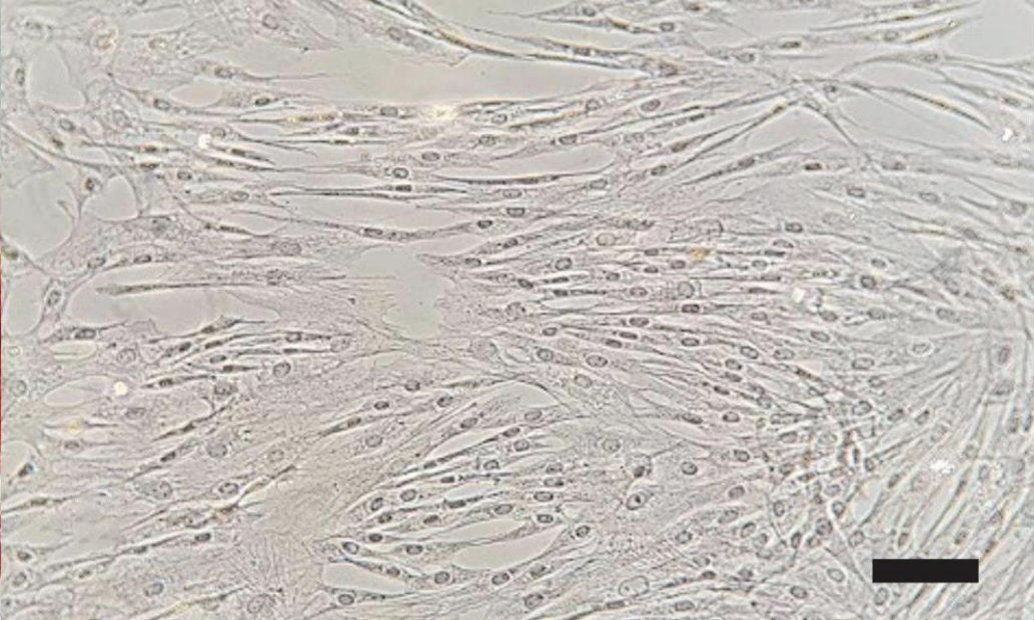
(B)

**Figure:**
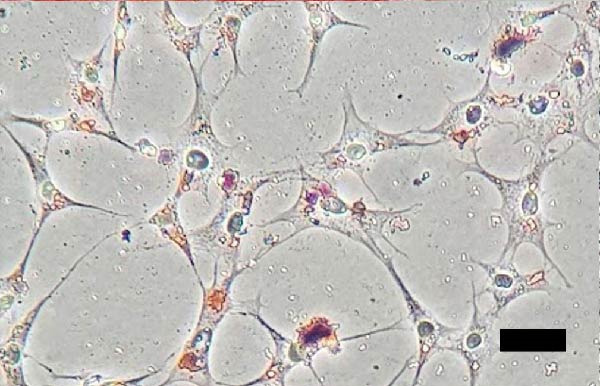
(C)

**Figure:**
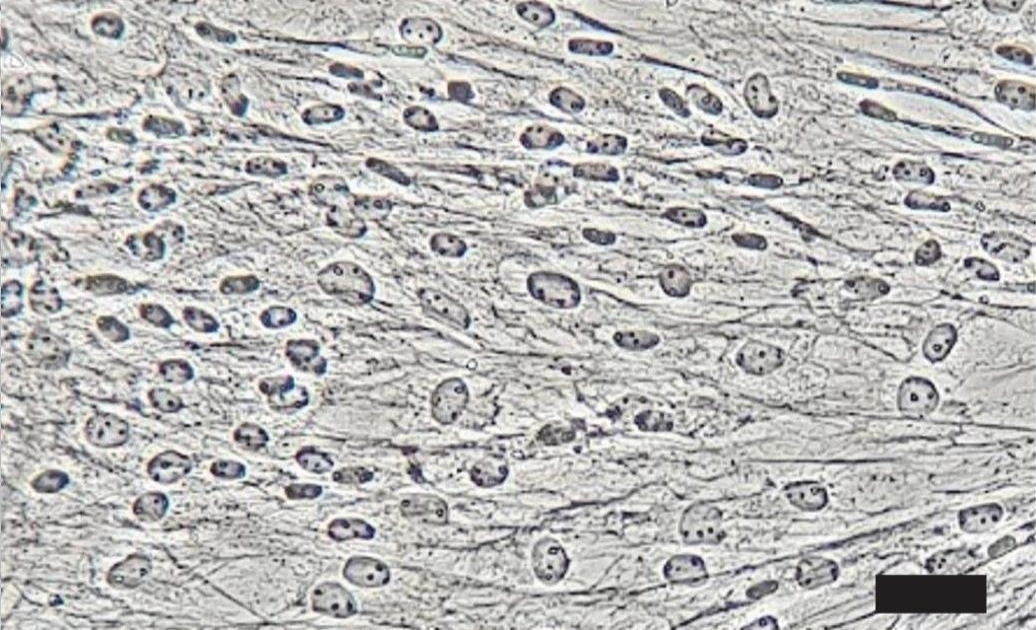
(D)

**Figure:**
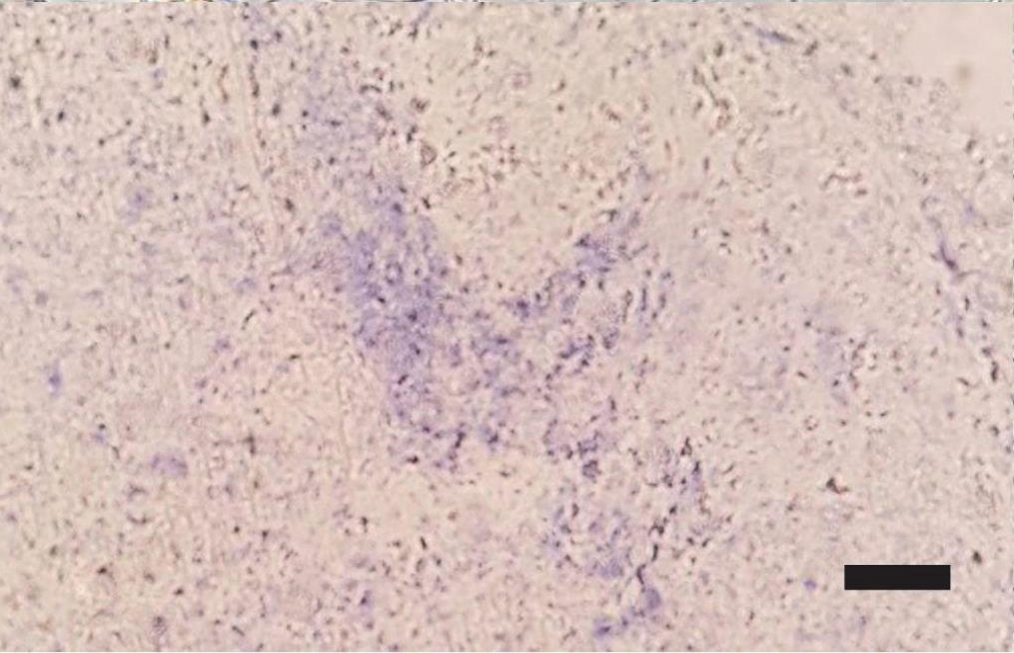
(E)

**Figure:**
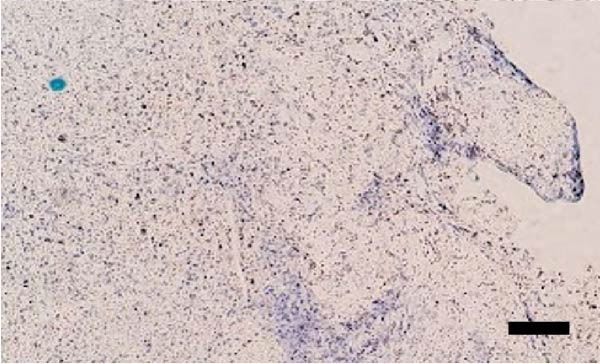
(F)

### 2.3. Gynecological Evaluation and Uterine Sampling

Cows were examined by vaginoscopy to assess and classify the presence of vaginal discharge (VD) [34] on the day of stem cell implantation (D0) and 30 days postimplantation (D30). For the vaginoscopic examination, the vulva was first cleaned with a paper towel, and a sanitized speculum was then gently inserted into the vaginal canal. Using a light source, the cervix and vaginal canal were carefully inspected, and any secretion present was categorized and recorded. VD was graded on a scale from 0 to 3, where 0 = mucus, 1 = mucus with flecks of pus, 2 = ≥50% purulent exudate, and 3 = hemorrhagic and/or purulent exudate, as adapted from previous studies [18, 34]. None of the animals selected for this study had VD.

For endometrial sample collection, an adapted cytological brush was attached to the tip of conventional artificial insemination (AI) equipment, which was covered by a disposable AI sheath and further protected by a sanitary sheath, as previously described [35, 36]. The apparatus was introduced via the cervix, and the cytobrush was rotated to collect cells from the uterine body. Immediately after sampling, the cytobrush was detached from the apparatus and gently rolled onto a clean microscope slide, using only half of its circumference to ensure an adequate quantity of cellular material remained on the untouched surface for gene transcription analysis. The slides were air‐dried and prepared for transport to the laboratory. The cytobrush was placed in a 2 mL cryotube containing 1 mL of RNA later buffer and stored at −80°C for subsequent processing. In the laboratory, the slides were stained using a commercial kit (Quick Panoptic, Laborclin, Pinhais, Brazil). Two hundred cells per slide were counted under an optical microscope (100× magnification), including PMN, mononuclear, and epithelial cells, in order to calculate the proportion of PMNs. Animals with a PMN count below 10% were considered free of uterine infection, as defined by Kasimanickam et al. [37], and were the ones selected for this study.

### 2.4. Microbiological Examination

Samples for microbiological examination were obtained using the same sampling method applied for cytology. The shaft of the gynecological brush was cut to facilitate immersion in Stuart transport medium (REMEL, Lenexa, USA) and subsequently placed in Styrofoam with disposable ice for preservation. All materials were processed in the Microbiology Laboratory of the University of São Paulo in accordance with established protocols [38].

### 2.5. Histopathology Examination

Uterine biopsies were performed on D0 and D30 using 65‐cm‐long stainless steel Yeoman biopsy forceps (Hauptner, Solingen, Germany), which were sterilized with disinfectant (CB30TA, Cravinhos, Brazil) and 70% alcohol after being washed with an iodopovidone antiseptic solution. The forceps were introduced into the uterus via the vagina, passing through the cervix into the uterine body at the intercornual ligament region. A uterine fragment, ~5 mm in length, 2 mm in height, and 2 mm in depth, was excised. The fragments were fixed in 10% formalin and placed in plastic cassettes for subsequent processing. Samples were prepared for microscopic evaluation according to the method described by Meira et al. [39]. Microscopic analysis was performed by a single pathologist who was blinded to the animals’ identities. Histopathological evaluation of the endometrium, as seen in Figure 4, was used to confirm the presence of endometrial degeneration, which was required for the classification of animals as repeat breeders.

Figure 4Glandular endometrial tissue of repeat breeder cows; the black arrow indicates fibrin around the gland (A). Connective tissue in the uterine parenchyma of repeat breeder cows (B).

**Figure:**
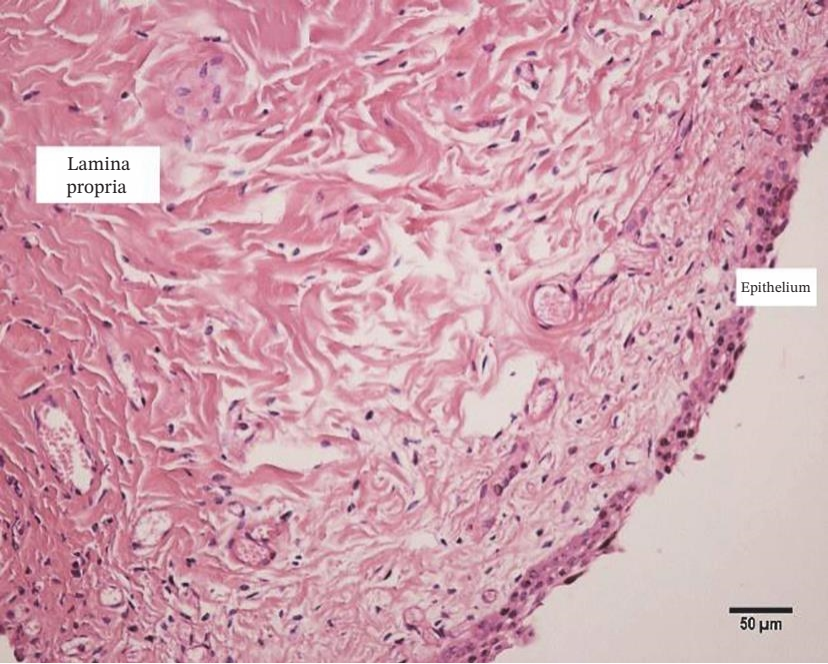
(A)

**Figure:**
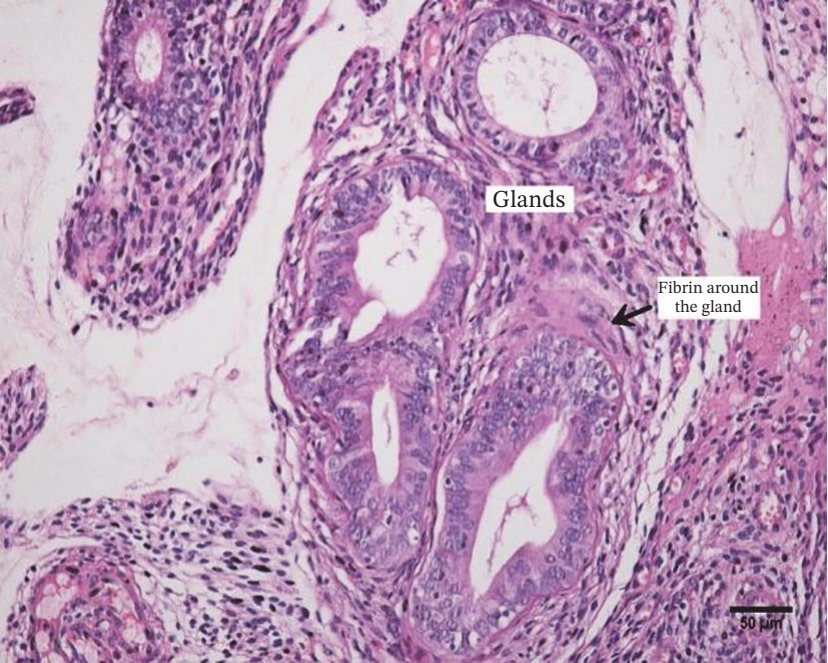
(B)

### 2.6. Ultrasound Examinations

Ovarian structures, the diameter of the uterine horns, the presence of content in the uterine lumen, the endometrial area, and uterine horn characteristics were assessed using cross‐sectional images obtained with a 5 MHz linear transducer in B‐mode (SIUI CTS‐900, Guangdong, China) on days 0 and 30. The evaluation of uterine horn vascularization was performed using Doppler mode (Mindray M5; Shenzhen, China) on a scale from 1 to 4 (1 = no vascularization and 4 = highly vascularized), as adapted from Ghinter and UTT [40], Ghinter [41], and Ribeiro [42]. Measurements were obtained from frozen images of the uterine horns, ~10 cm from the point of uterine bifurcation. The diameter was defined as the vertical dimension from serosa to serosa through the center of the organ.

Ultrasound examination (SIUI CTS‐900, 5 MHz linear transducer, Guangdong, China) was conducted 30 days after fixed‐time AI (FTAI) for pregnancy diagnosis. Visualization of the embryonic vesicle and detection of the embryo were used as positive criteria for confirming pregnancy.

### 2.7. Cell Implantation

The cryotubes were thawed in a water bath at 36°C and then centrifuged at 161 × *g* for 10 min. Following centrifugation, the supernatant, consisting of the cell preservation solution (PBS), was discarded, leaving the MSC pellet. Next, 20 mL of 0.9% sodium chloride (NaCl) physiological solution was added to the tubes to dissolve the pellet and dilute the cells.

The MSC implantation procedure was performed after cleaning the perineal area. The operator, wearing a sterile insemination glove, introduced a disposable insemination pipette through the cervix into the uterine body. To prevent contamination, the gloved hand was placed over the tip of the pipette during its introduction into the vagina. The pipette was connected to a syringe containing 1 × 10^6^ cells/mL, diluted in 20 mL of 0.9% NaCl, via a sterile rubber connector. The plunger of the syringe was slowly depressed, administering 10 mL of the cell suspension at 20 different points (sites of administration), spaced 1 cm apart, along a horizontal line at the end of a uterine horn. Immediately after, a second syringe containing 3 mL of 0.9% NaCl was attached to the sterile pipette and infused to ensure the complete delivery of the cell suspension. The pipette was then slowly withdrawn from the vagina.

### 2.8. Reproductive Management

At the end of the final collection (D30), 150 μg of D‐cloprostenol (PGF2α analog; Croniben, Biogénesis‐Bagó, Curitiba, Brazil) was administered intramuscularly to the 10 animals to regress the corpus luteum (CL), allowing them to enter natural estrus and be inseminated.

### 2.9. RNA Isolation and cDNA Synthesis

Total RNA was extracted from cytobrush samples of the 10 cows, both untreated and MSC‐treated. Uterine cells in tubes were pelleted by centrifugation at 2000 × *g* for 10 min at 4°C. The supernatant (600 μL) was discarded, and 600 μL of 1% *β*‐mercaptoethanol was added before total RNA was extracted according to the commercial kit protocol (PureLink RNA Mini Kit, Thermo Fisher Scientific, Carlsbad, CA). The purity and quantity of RNA were assessed using a NanoDrop 2000 spectrophotometer (Thermo Fisher Scientific, USA). The RNA was treated with DNase I (Invitrogen, Thermo Fisher Scientific, USA) before reverse transcription (RT) to remove possible genomic DNA contamination.

For cDNA synthesis, the Anchored Oligo primer (dT) 23 (Sigma–Aldrich, USA) was incubated with the treated total RNA at 70°C for 10 min, as described by the manufacturer. The RT reaction was performed using the High‐Capacity cDNA kit (Applied Biosystems, USA) according to the protocol described by the manufacturer, in a final volume of 20 μL/reaction, using the following conditions: 25°C for 10 min, 37°C for 120 min, and 85°C for 5 min. The synthesized cDNA was subsequently quantified by spectrophotometry (NanoDrop 2000 spectrophotometer; Thermo Fisher Scientific, USA) to assess the RT reaction efficiency and was stored at −80°C until quantitation assays were conducted.

### 2.10. Quantitative Real‐Time Polymerase Chain Reaction (qRT‐PCR)

qRT‐PCR was used to assess the relative abundance of mRNAs in the cytobrush samples and was performed by an operator blinded to the cytology results. The Perfecta SsoFast EvaGreen Supermix Kit (Bio‐Rad Laboratories Inc., USA) was used according to the manufacturer’s instructions to perform qRT‐PCR analysis of the relative abundance of the mRNA associated with regulation of cell proliferation in the uterus: estrogen receptor α (ESR1), estrogen receptor β (ESR2), P4 receptor (PGR), growth factors that regulate cellular proliferation–epidermal growth factor receptor (EGFR), heparin‐binding EGF‐like growth factor (HBEGF), patched homolog 2 (PTCH2), collagen type IV α1 (COL4A1), cytokine expression (IL‐1β and IL‐8), glyceraldehyde‐3‐phosphate dehydrogenase (GAPDH), and β‐actin (ACTB). Briefly, 10 μL of Perfecta SsoFast EvaGreen master mix for iQ (2x), 1 μL of the appropriate primer set, as documented in Table 1, at 10 μM, 250 ng/μL of cDNA template, and 4 μL of water were added for a final reaction volume of 20 μL. The reaction was performed in the Bio‐Rad CFX96 detection system (Bio‐Rad Laboratories Inc., Singapore) using the following program: 1 cycle at 95°C for 5 s, 39 cycles at 95°C for 5 s, and 60°C for 20 s, and a melting curve standardized from a variable annealing temperature ramp from 65 to 95°C with an increase of 0.5°C every 5 s. Samples were amplified in duplicate, and a melting curve was completed after each PCR reaction to ensure fluorescence quantification was specific to a single PCR product. Both no‐template and no‐reverse transcriptase controls were utilized to verify the DNA‐free status of the negative control samples.

**Table 1:** Primer sequences for quantitative real‐time polymerase chain reaction (qRT‐PCR) amplification of mRNA.

Gene	Gene ID	Primer direction	Primer sequence (5’ ‐3’)	Amplicon length, bp	OBS
P4 receptor	PGR	Forward	GCCGCAGGTCTACCAGCCCTA	199	Regulation of cell proliferation in the uterus
		Reverse	GTTATGCTGTCCTTCCATTGCCCTT		
Estrogen receptor α	ESR1	Forward	CAGGCACATGAGCAACAAAG	82	Regulation of cell proliferation in the uterus

		Reverse	TCCAGCAGCAGGTCGTAGAG		
Estrogen receptor β	ESR2	Forward	TCACGTCAGGCACGCCAGTAAC	155	Regulation of cell proliferation in the uterus
		Reverse	CACCAGGTTGCGCTCAGACCC		
Patched 2	PTCH2	Forward	CATCCTGCTGCTGTGTACTT	87	Cell proliferation Embryogenesis
		Reverse	ATCGCCAGGACCAGTACTAT		
Epidermal growth factor receptor	EGFR	Forward	ATGCTCTATGACCCTACCAC	178	Growth factor cell
		Reverse	TTCCGTTACAAACTTTGCCA		
Heparin‐binding EGF‐like growth factor	HBEGF	Forward	CATCCACGGAGAATGCAAATAC	181	Growth factor cell
		Reverse	CAGCAGACAGACGGATGATAG		
Collagen, type IV, α 1	COL4A1	Forward	CACGGCTACTCTTTGCTCTAC	102	Membrane component
		Reverse	GAAGGGCATGGTACTGAACTT		
Interleucin −1 Beta	IL‐1β	Forward	AGCATCCTTTCATTCATCTTTGAAG	—	Cytokines
		Reverse	CCTGTCATCTTCGAAACGTCCTCCGA		
Interleucin ‐ 8	IL‐8	Forward	TGCTTTTTTGTTTTCGGTTTTTG AACAGGCACTCGGGAATCCT	—	Cytokines
		Reverse	TAATCTTGCAACCCTCACCTGCTGGC		
Interleucin ‐ 10	IL‐10	Forward	AGAACCACGGGCCTGACAT AGCTCACTGAAGACTCTCTTCACCTT	—	Cytokines
		Reverse	TTCTGCCCTGCGAAAACAAGAGCAA		
Glyceraldehyde‐3‐phosphate dehydrogenase	GAPDH	Forward	GCCATCAATGACCCCTTCAT	69	Reference genes
		Reverse	TGCCGTGGGTGGAATCA		
Actin β	ACTB	Forward	GGATGAGGCTCAGAGCAAGAGA	77	Reference genes

The changes in gene transcription were calculated by the 2^-ΔΔCT^ method [43] using the selected reference genes (β‐actin and GAPDH). The first control sample was expressed as 1.0 by this equation, and all other samples were calculated in relation to this value. Afterward, the results in the control (untreated) were averaged, and all other outputs were divided by the mean value of the relative abundance in the control group to yield the fold change of the genes of interest compared to the control group [44].

### 2.11. Statistical Analysis

The statistical analysis was performed using the GraphPad Prism 8 software. The statistical premises of the parametric analysis were verified by the Shapiro–Wilk and Bartlett tests; the null hypothesis was rejected in both; thus, parametric analysis guidelines were used to infer more accurately the results. In order to prove the difference between the treatments, an analysis of variance (ANOVA) was used, reaffirmed by the two‐way *t*‐test. Pregnancy per AI was analyzed using the chi‐square test. The Mann–Whitney test was used for the analysis of relative abundances of mRNA. A significant level of 5% was considered for all tests performed.

## 3. Results

After evaluating 10 cows, a total of nine were selected, four of them primiparous animals. These animals had undergone an average of six AIs, either following natural estrus or induced through hormonal protocols, and were consequently classified as repeat breeders.

### 3.1. Collection and Cultivation of MSCs

Cells from all three sources displayed fibroblast‐like morphology and adhered to plastic, consistent with MSC characteristics, as observed in Figure 2. Bone marrow–derived cells adhered after ~5 days and reached ~ 80% confluence within 20 days.

Chondrogenic differentiation was confirmed after 21 days by Alcian Blue and Toluidine Blue staining, which revealed a proteoglycan‐rich extracellular matrix and chondrocyte‐like cells, as shown in Figure 3E.

Osteogenic differentiation was also observed after 21 days and confirmed by Alizarin Red staining of calcium deposits, whereas control cells showed no mineralization, as shown in Figure 3A.

Adipogenic differentiation appeared as small foci at the plate periphery. Despite some cell death following induction, adipogenesis was confirmed by the morphological shift from spindle‐shaped to polygonal cells, observed in Figure 3C, and the presence of cytoplasmic lipid droplets stained with Oil Red O.

### 3.2. Immunophenotypic Characterization

The immunophenotypic analysis of the bovine bone marrow–derived cells demonstrated a profile consistent with the phenotype of MSCs. The expression of the characteristic positive MSC markers was confirmed, as shown in the histograms in Figure 1. It was observed that 47% of the cells were positive for CD90 (Figure 5D), 75.6% expressed CD105 (Figure 5E), and 74.2% were marked for CD73 (Figure 5F). Each marker exhibited a clear fluorescence shift relative to the isotype control, confirming specific staining and indicating the presence of a mesenchymal phenotype. In contrast, the expression of hematopoietic and immunological markers expected to be negative in the standard MSC phenotype was minimal, as shown in Figure 2. Only 0.97% of the cells were positive for CD34 (Figure 6D), 0.69% for CD45 (Figure 6E), and 0.58% for MHC‐II (Figure 6F). These findings demonstrate low or absent expression of markers associated with hematopoietic lineages and antigen‐presenting cells, reinforcing the typical nonimmunogenic nature of MSCs. Taken together, the data confirm that the analyzed cell population displays the immunophenotypic profile expected for MSCs, characterized by positive expression of CD73, CD90, and CD105, and absence of CD34, CD45, and MHC‐II. This profile, combined with the morphology observed in the scatter gates, validates the mesenchymal identity of the isolated cells. In contrast, the expression of hematopoietic and immunological markers expected to be negative in the standard MSC phenotype was minimal, as shown in Figure 2. Only 0.97% of the cells were positive for CD34 (Figure 6D), 0.69% for CD45 (Figure 6E), and 0.58% for MHC‐II (Figure 6F). These findings demonstrate low or absent expression of markers associated with hematopoietic lineages and antigen‐presenting cells, reinforcing the typical nonimmunogenic nature of MSCs. Taken together, the data confirm that the analyzed cell population displays the immunophenotypic profile expected for MSCs, characterized by positive expression of CD73, CD90, and CD105, and absence of CD34, CD45, and MHC‐II. This profile, combined with the morphology observed in the scatter gates, validates the mesenchymal identity of the isolated cells.

Figure 5Phenotypic analysis of positive mesenchymal stem cell (MSC) markers in bovine cells by flow cytometry. (A–C) Dot plots (FSC × SSC) showing the gated cell population corresponding to a mesenchymal phenotype, representing 71.2%, 67.5%, and 66.7% of total cells, respectively. (D–F) Fluorescence histograms illustrating the expression of the characteristic positive MSC markers: CD90 (47.0%), CD105 (75.6%), and CD73 (74.2%). A clear fluorescence shift relative to isotype controls is observed, confirming specific labeling.

**Figure:**
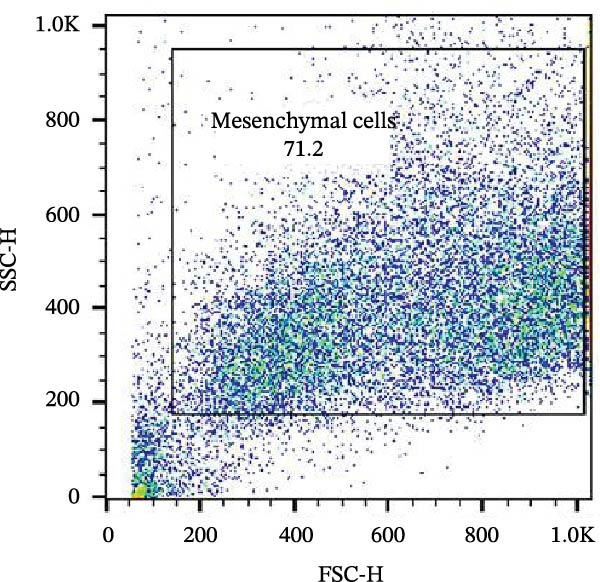
(A)

**Figure:**
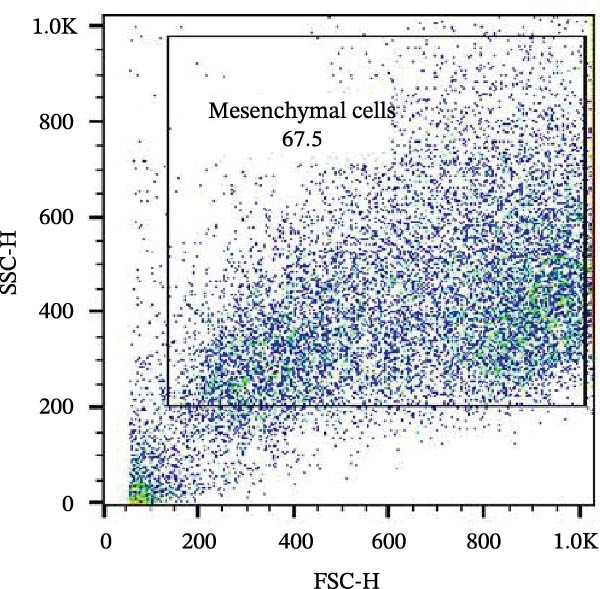
(B)

**Figure:**
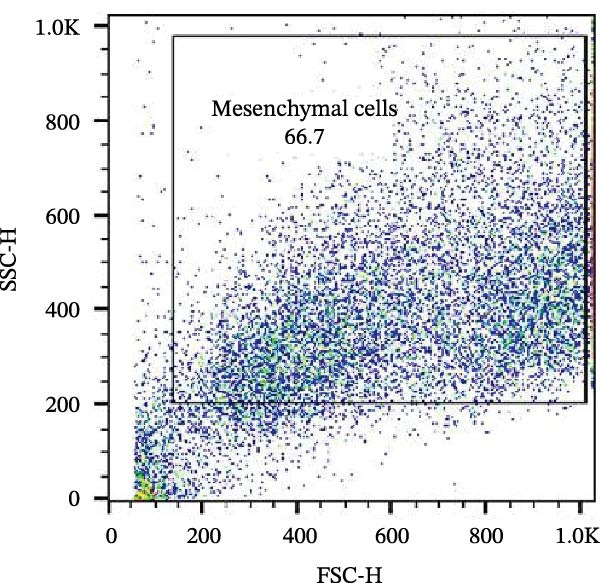
(C)

**Figure:**
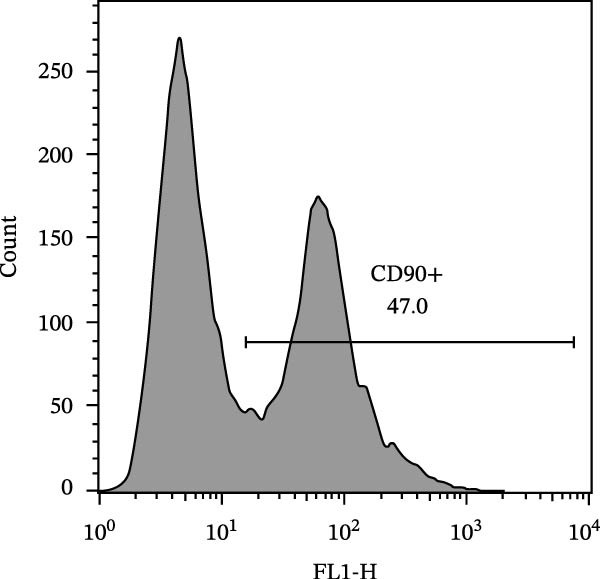
(D)

**Figure:**
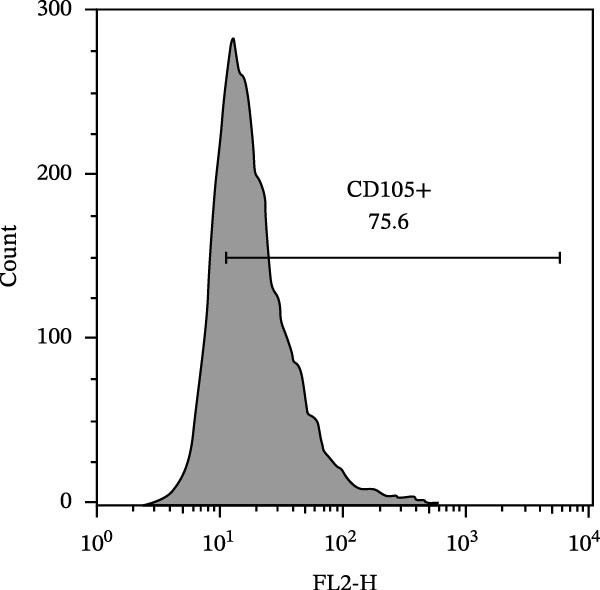
(E)

**Figure:**
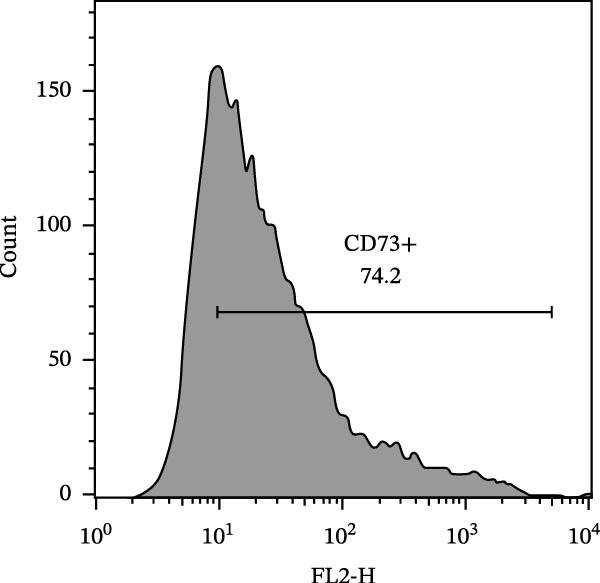
(F)

Figure 6Analysis of negative markers for bovine MSCs by flow cytometry. (A–C) Dot plots (FSC × SSC) showing the gated cell population with morphological features consistent with MSCs, representing 71.3%, 69.1%, and 70.5% of the acquired cells. (D–F) Histograms showing minimal expression of hematopoietic and immunological markers: CD34 (0.97%), CD45 (0.69%), and MHC‐II (0.58%). The absence of significant fluorescence confirms the expected MSC profile, characterized by low immunogenicity and lack of hematopoietic features.

**Figure:**
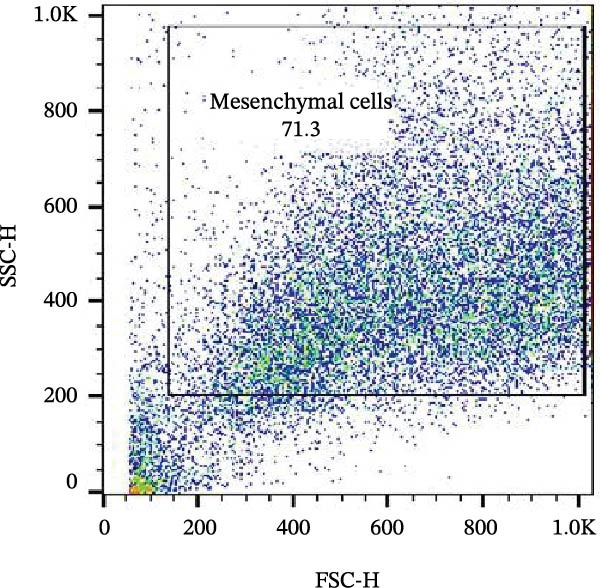
(A)

**Figure:**
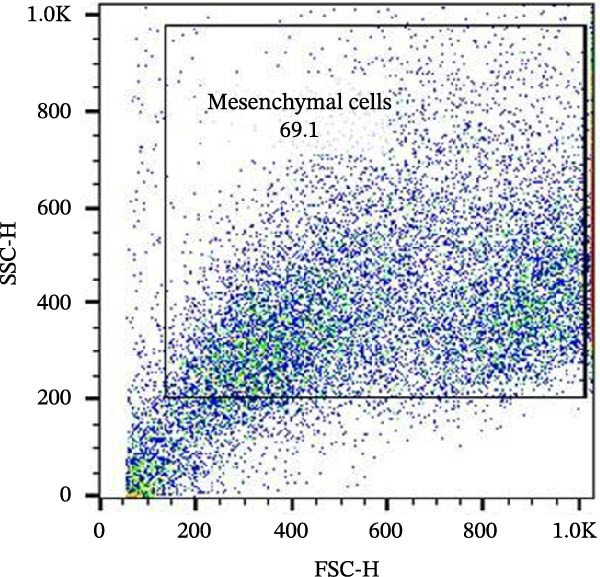
(B)

**Figure:**
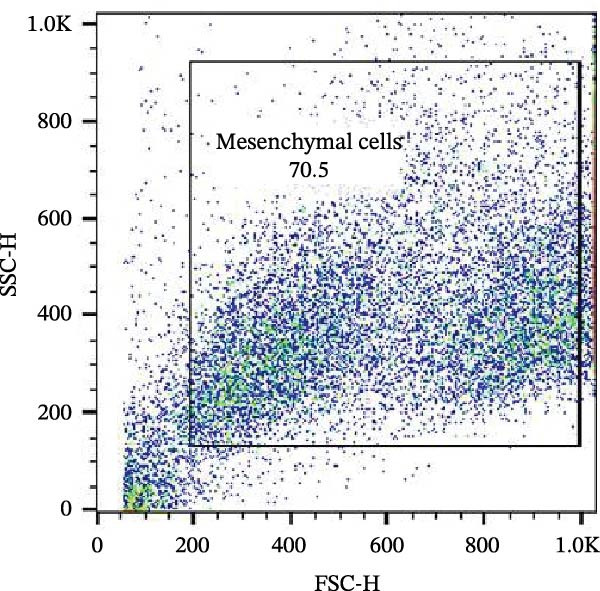
(C)

**Figure:**
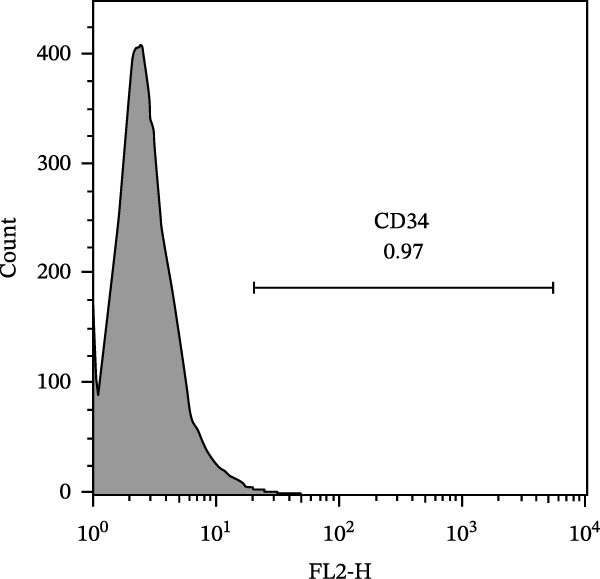
(D)

**Figure:**
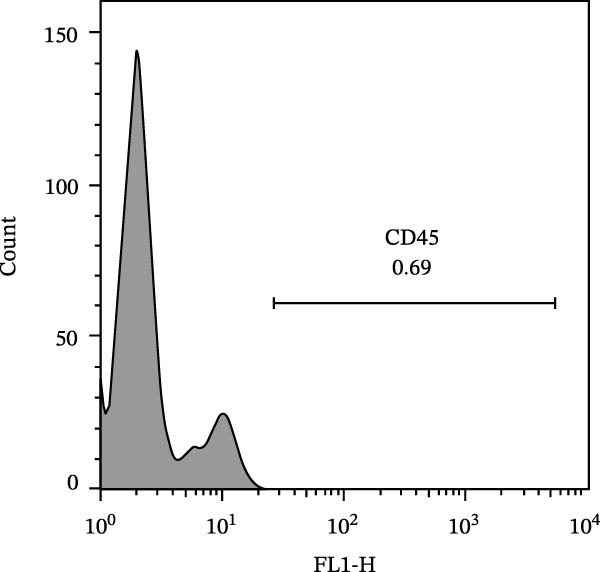
(E)

**Figure:**
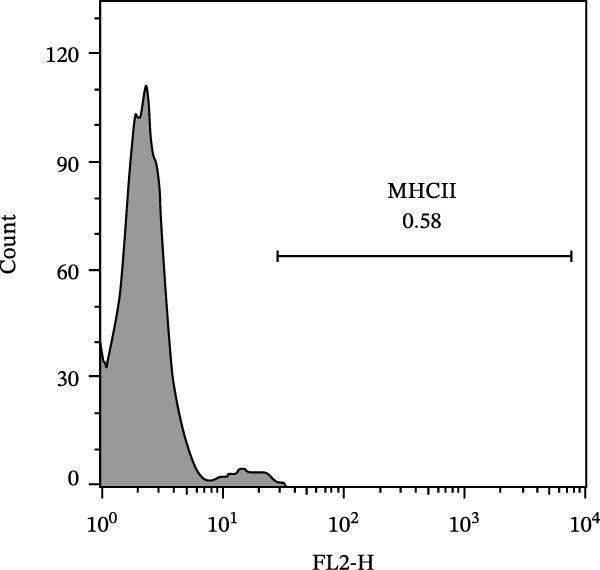
(F)

### 3.3. Microbiological Examination

The results of the microbiological examination did not indicate a high bacterial load. Only bacilli were isolated and identified. There was no growth of *Fusobacterium necrophorum* or Trueperella pyogenes, ruling out bacterial infection as a likely cause of reproductive failure.

### 3.4. Ultrasound Examinations

The dimensions and color Doppler findings of the right and left uterine horns are presented in Table 2.

**Table 2:** Mean values and standard deviation of the variables body condition score (BCS), left uterine horn, right uterine horn, color Doppler scale, and endometrial cytology in 9 animals subjected to stem cell treatment, assessed in two different periods with a 30‐day interval.

Variables analysed	Day 0 Mean ± SD	Day 30 Mean ± SD
BCS, (1–5)	4,0 ± 0,27	3,75 ± 0,28
Left uterine horn, (cm)	1,73 ± 0,31	1,89 ± 0,23
Right uterine horn, (cm)	1,66 ± 0,29	2,18 ± 0,35 ^∗^
Color doppler signs (1–4)	1,00 ± 1,33	2,0 ± 0,71
Endometrial cytology, %	2,00 ± 6,00	0,0 ± 0,44

^∗^Significant difference between moments Day 0 and Day 30 through the paired Student’s test (*p* < 0.05), in the same line.

Cows on D0 had smaller diameters of the right and left uterine horns (*p* > 0.05) than on D30. No significant difference was observed between the right and left uterine horns (*p* < 0.05) at any of the time points evaluated, both on D0 and D30. However, when comparing D0 and D30, an increase in the vascularization of the endometrial mucosa and thickening of the uterine wall were noted following the inoculation of MSCs, indicating a positive response to the treatment.

Eight cows from the study were inseminated but did not have a positive pregnancy (0/8).

### 3.5. Gynecological Evaluation and Uterine Sampling

The proportion of PMN cells in the uterus is shown in Table 1. There was no difference (*p* < 0.05) in the proportion of PMN cells on D0 (2%) and D30 (0%).

### 3.6. Histopathology Examination

Histopathological analysis of uterine tissues, shown in Figure 7, at D0 revealed predominantly dense and diffuse fibrosis, observed in eight of nine samples (88.9%). Among these, four samples exhibited dense, diffuse fibrosis with mild vacuolization of the columnar endometrial epithelium, while three additional samples also presented dilated and congested blood vessels, mild edema, and occasional eosinophilic leukocytes. Secretory phase characteristics—including luminal secretion and glandular dilation—were identified in one sample. One fragment showed mixed fibrosis patterns, with both loose and dense areas, and one sample contained insufficient tissue for complete assessment.

Figure 7Photomicrography of endometrial mucosa of repeat breeder cows. (A) First biopsy, cow 39. Diffuse dense fibrosis of endometrial mucosa. (B) Second biopsy, 30 days after mesenchymal stem cell therapy, cow 39. Mucosa was replaced by soft stroma with few gland units and an increased number and size of vessels. (C) Image B at higher magnification. (D) First biopsy, cow 73. Dense diffuse fibrosis. (E) Second biopsy of cow 100. Lamina propria was replaced by soft tissue with an increased number of vessels. (F) Second biopsy of cow 104. Few endometrial glands in soft tissue with an increased number and size of vessels. Hematoxylin and eosin.

**Figure:**
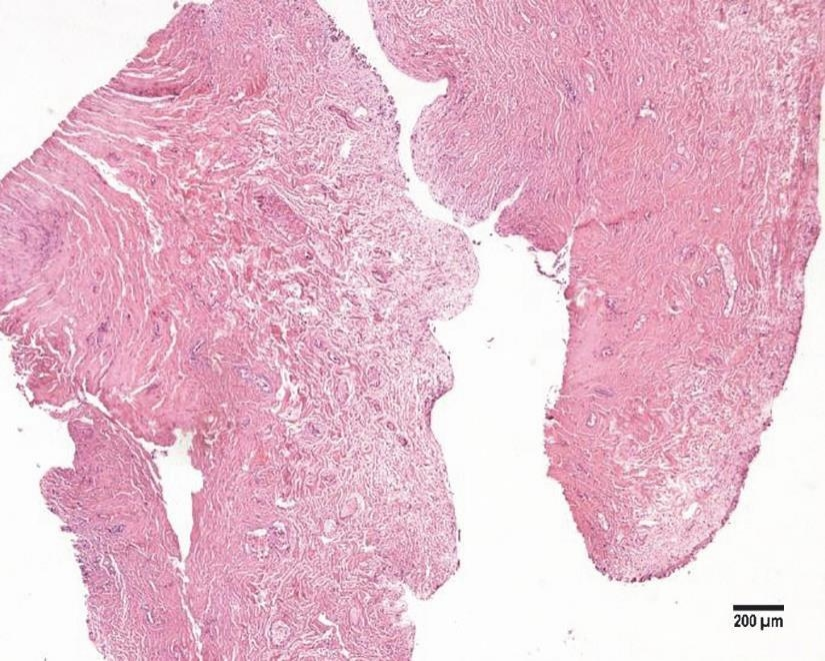
(A)

**Figure:**
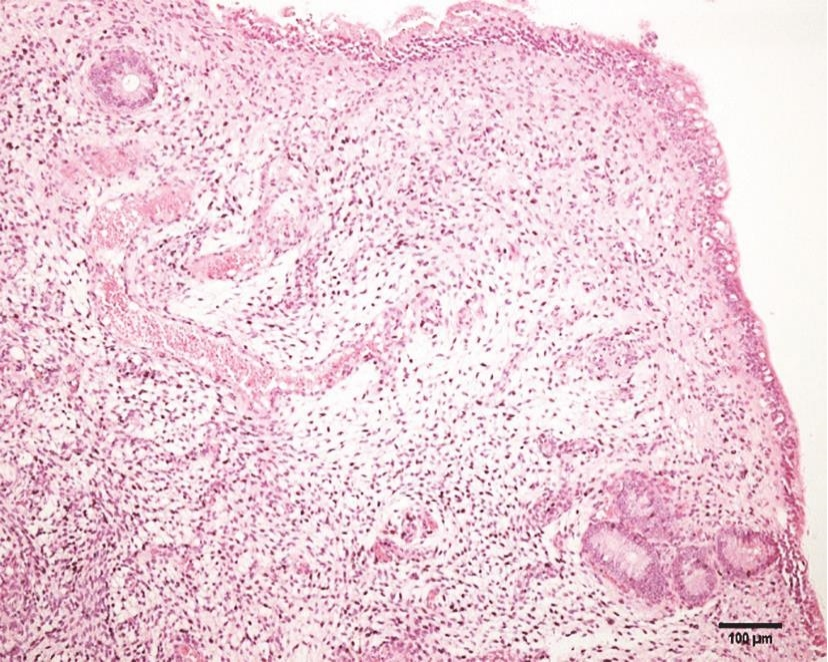
(B)

**Figure:**
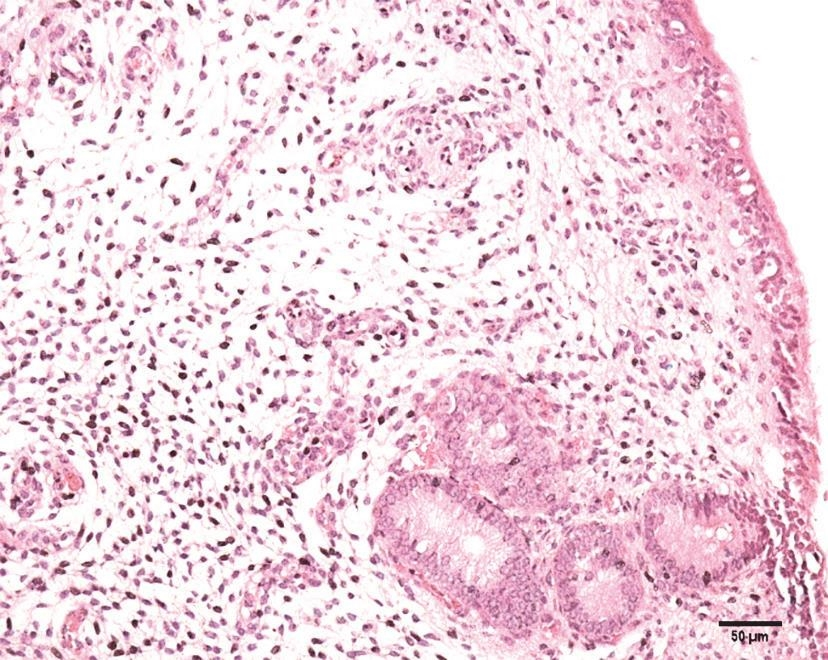
(C)

**Figure:**
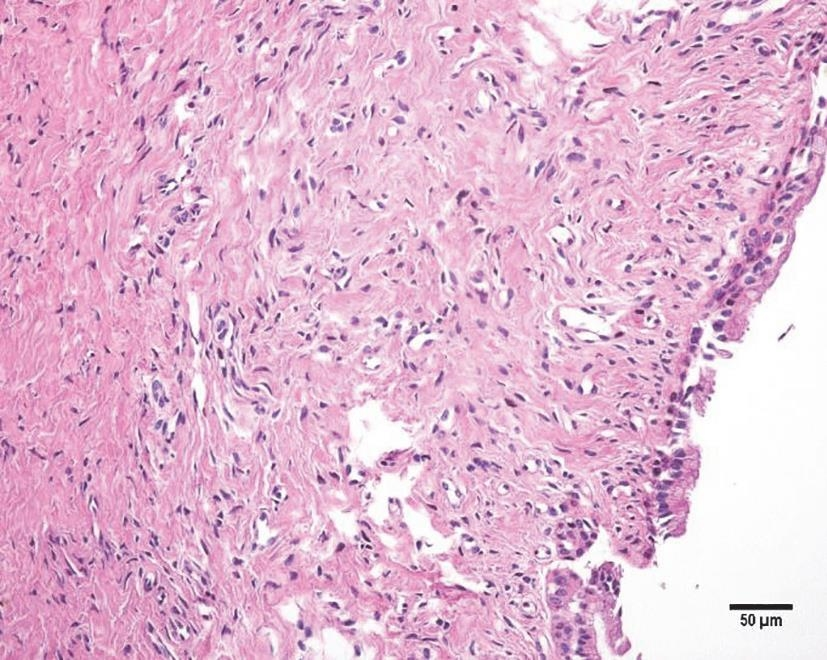
(D)

**Figure:**
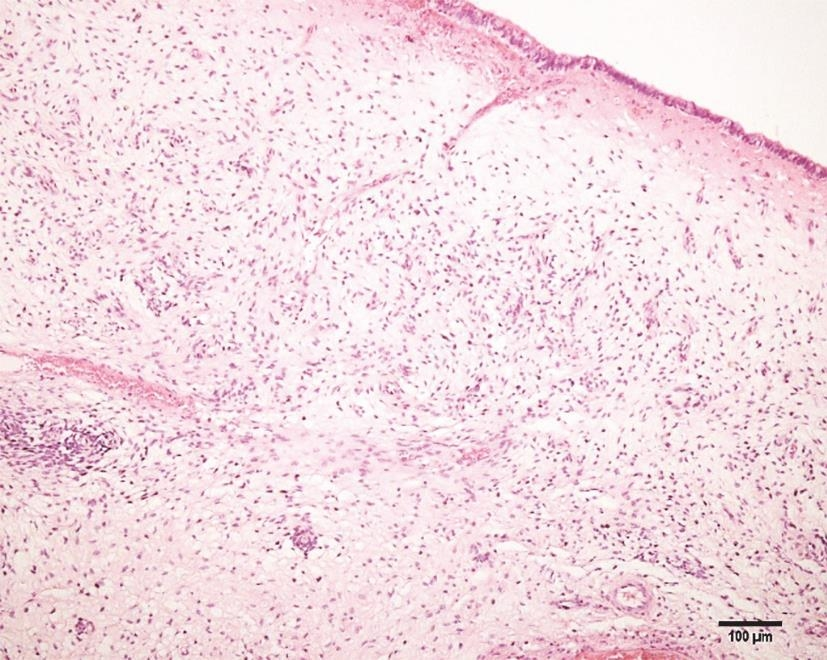
(E)

**Figure:**
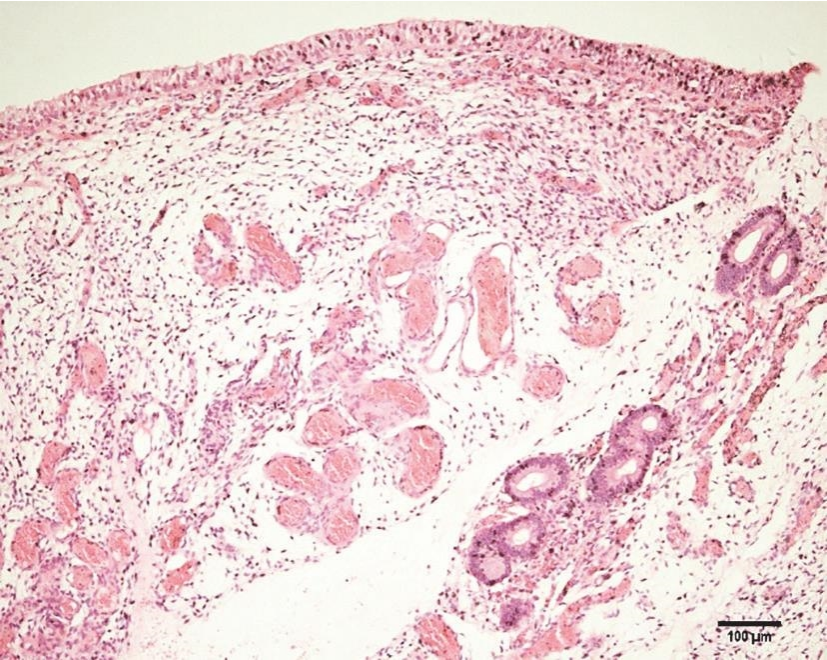
(F)

At D30 (after infusion), the mucosa showed marked remodeling, characterized by increased vascularization, enlargement and proliferation of endometrial glands, and neovascularization within the superficial lamina propria. The connective tissue in this region appeared loose rather than densely fibrotic, suggesting partial reversal of fibrosis. No significant difference in BCS was observed between D0 and D30 (*p* > 0.05; shown in Table 2).

### 3.7. QRT‐PCR

**Figure 8:**
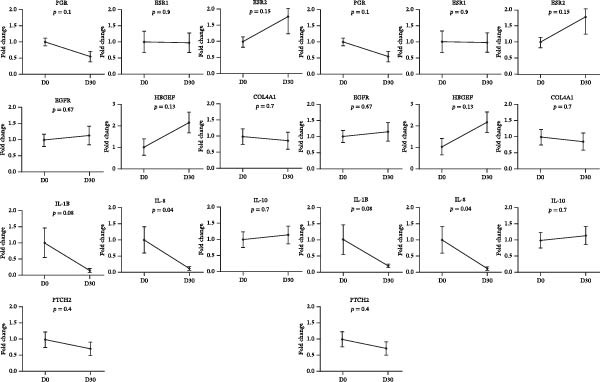
Relative abundance of mRNA transcription in uterine endometrial tissues of repeat breeder cows treated and untreated with mesenchymal stem cells.

The relative abundance of PGR, ESR1, ESR2, PTCH2, EGFR, HBEGF, COL4A1, IL‐1β, IL‐8, and IL‐10 mRNA is depicted in Figure 8. There was no difference in the control (β‐actin and GAPDH) Ct values between days D0 and D30 (*p* = 0.9; 25.1 and 25.0 in cows from D30 and D0, respectively). The consistency of the abundance of β‐actin and GAPDH mRNA confirms their suitability as reference genes. Relative abundance of mRNA transcripts for PGR, ESR1, ESR2, EGFR, HBEGF, COL4A1, IL‐10, and PTCH2 did not change among collections (*p* < 0.05). The relative abundance of mRNA transcripts for IL‐1β and IL‐8 was greater (*p* = 0.08 and *p* = 0.04, respectively) in D0 than in D30.

## 4. Discussion

Endometrial degeneration is characterized by an excessive proliferation of connective tissue surrounding endometrial glands [45], a phenomenon corroborated in the present study, where dense connective tissue around the glands was observed, potentially impairing their function. When comparing pre‐ and postcell therapy data, a significant change in uterine diameter was observed in both uterine horns. Several hypotheses may explain this phenomenon. First, hormonal influences cannot be ruled out, as physiological uterine alterations naturally occur during the reproductive cycle [46]. Second, delayed uterine involution may be a contributing factor. Slama et al. [47] demonstrated that uterine infections can predispose cattle to increased uterine diameter and prolonged puerperal involution. Mateus et al. [48] further reported that delayed involution negatively impacts reproductive performance. However, in the present study, this hypothesis was dismissed, as cytological evaluation conducted 30 days posttreatment did not reveal any uterine disease indicators.

An alternative explanation for the increased uterine diameter relates to findings by Gutierrez [49], who reported that MSC therapy in pulmonary fibrosis initially induced an inflammatory response, followed by tissue repair and fibrosis reduction. However, our findings indicate that MSC therapy reduced IL‐1β and IL‐8 expression after 30 days, suggesting a shift toward a less inflammatory uterine environment. Inflammation plays a critical role in uterine receptivity and fertility, as excessive immune activation can impair embryo implantation and endometrial function. Previous research has demonstrated that elevated levels of proinflammatory cytokines, particularly IL‐1β and IL‐8, are associated with lower pregnancy per AI rates in cattle [17]. This correlation likely results from the detrimental effects of chronic inflammation on endometrial integrity, vascularization, and immune tolerance, all of which are essential for successful conception and pregnancy maintenance.

All animals in this study exhibited normal estrous cyclicity, as they were subjected to AI protocols. Ultrasound evaluations confirmed the presence of key reproductive structures, including vascularized CL, which is essential for estrous identification [49, 50]. Endometrial alterations can influence ovarian function and disrupt cyclicity [51]; however, Fu et al. [52] demonstrated a positive correlation between MSC therapy and ovarian functionality. In the present study, no adverse effects on ovarian function were observed, as all animals entered estrus and were inseminated after the experiment. Despite this, pregnancy was not achieved. This outcome may reflect factors not directly assessed in this study, such as the optimal timing of insemination relative to tissue repair, the possibility that the 30‐day interval was insufficient for complete functional recovery, or undetected hormonal or molecular abnormalities that continued to compromise fertility. As a pioneering trial, the single intrauterine dose of 10^6^ cells was chosen based on feasibility and prior pilot data, but further research is necessary to establish the most effective dose, number of applications, and interval between therapy and breeding.

Although MSCs are widely recognized for their regenerative potential, particularly in tissue repair, their application in repeat breeder cows remains unexplored. In mares with endometritis, MSC therapy has shown promising results in endometrial regeneration [53, 54]. Gattegno‐Ho et al. [53] observed that MSCs actively participate in tissue remodeling and healing processes, while Vassena et al. [45] reported that MSC therapy enhances the proliferation of endogenous progenitor cells, thereby increasing tissue regeneration capacity [55].

Studies indicate that the treatment of endometritis in mares and cows employs cell doses ranging from 10^6^ to 10^8^ [56, 57]. However, there is still no scientific consensus regarding the standardization of the optimal cell number for treatment, and protocols are generally based on the outcomes of pilot studies. In our study, a single dose of 10^6^ cells was administered. Based on the interpretation of our results, it is possible to suggest that higher doses may be more effective, as reported in other studies in which 10^7^ cells [56] and 2 × 10^7^ cells [57] were applied, demonstrating a proven anti‐inflammatory effect when administered via the intrauterine route.

Our study employed only a single application, and research addressing multiple applications remains scarce. This is an important point to be explored in future studies to assess whether repeated administrations favor the maintenance of the anti‐inflammatory effect due to the paracrine action of the cells. In buffaloes with endometritis, treatment with two applications yielded favorable clinical results [57], suggesting that further studies are warranted to determine whether this approach could also improve pregnancy rates.

When considering other regenerative therapies, platelet‐rich plasma (PRP) has demonstrated modulatory effects on uterine inflammation, improving the inflammatory process in cows [58] and mares [59]. However, studies combining MSCs with PRP indicate that PRP may induce undesirable effects on endometrial cells [60]. For example, in an in vitro study using conditioned medium derived from MSCs isolated from equine umbilical cord Wharton’s jelly, a protective effect on endometrial cells was observed even after LPS challenge. The conditioned medium prevented the deleterious effects of LPS, maintained cell viability, and suppressed PGE‐2 production, suggesting its therapeutic potential in modulating endometrial inflammation.

Conversely, PRP exerted detrimental effects on endometrial cells, including reduced cell viability, increased production of reactive oxygen species (ROS), and induction of PGE‐2 secretion, without a significant effect on IL‐10 production [61]. These findings indicate that, in this context, PRP alone may not be beneficial for the treatment of endometrial inflammation.

The limitations of this study include the complexity of MSC isolation and expansion, which require specialized personnel and infrastructure, as well as the logistical and financial constraints inherent to in vivo trials in large animals, where sample sizes and observation periods are limited. Additionally, although a separate control group was not included, a paired‐sample design was intentionally used so that each cow served as its own control, allowing direct comparison of pre‐ and posttreatment uterine conditions while minimizing interindividual variation. This approach effectively demonstrated histological and molecular regeneration.

## 5. Conclusions

This study provides histological and molecular evidence that intrauterine MSC therapy promotes endometrial regeneration in repeat breeder cows characterized by uterine fibrosis. Thirty days after treatment, the replacement of dense fibrotic tissue by loose connective tissue, increased vascularization, and reduced expression of proinflammatory cytokines (IL‐1β and IL‐8) collectively indicated a favorable remodeling of the uterine environment. These findings confirm the regenerative potential of MSCs in bovine endometrium and support their use as a novel therapeutic tool for improving uterine health.

Nevertheless, the absence of pregnancies following treatment highlights that endometrial regeneration alone may not guarantee restoration of fertility. As a pioneer trial, this work demonstrates feasibility but also reveals key barriers, including the need for standardized cell dosage, multiple applications, and long‐term evaluation.

Future studies should focus on protocol optimization and expand the follow‐up period to determine whether the histological regeneration observed here translates into improved conception rates. Despite its limitations, this study establishes foundational evidence that MSC therapy can induce structural and molecular regeneration of the bovine endometrium and opens new perspectives for regenerative approaches in reproductive management of repeat breeder cows.

**Nomenclature**
ACTB: β‐Actin
AI: Artificial insemination
ANOVA: Analysis of variance
BCS: Body condition score
cDNA: Complementary DNA
COL4A1: Collagen type IV α1
D0: Day 0, untreated
D30: Day 30, treated
DMEM/F12: Dulbecco’s modified eagle medium/nutrient mixture F‐12
DNase I: Deoxyribonuclease I
EGFR: Epidermal growth factor receptor
ESR1: Estrogen receptor α
ESR2: Estrogen receptor β
GAPDH: Glyceraldehyde‐3‐phosphate dehydrogenase
HBEGF: Heparin‐binding EGF–like growth factor
IL‐1β: Interleukin‐1 beta
IL‐8: Interleukin 8
mRNA: Messenger RNA
MSCs: Mesenchymal stem cells
PBS: Phosphate‐saline buffer
PGR: P4 receptor
PGF2α: Prostaglandin F2α
PMN: Polymorphonuclear cells
PTCH2: Patched homolog 2
qRT‐PCR: Quantitative real‐time polymerase chain reaction
RB: Repeat‐breeding
RT: Reverse transcription
TAI: Timed artificial insemination
VD: Vaginal discharge
CL: Corpus luteum.

## Author Contributions

Bruno Leonardo Mendonça Ribeiro conducted fieldwork and was a major contributor in writing the manuscript. Joice Fülber developed stem cells. Mario Augusto Reyes Aleman conducted fieldwork. Luiz Francisco Machado Pfeifer performed the study of immunology. Jéssica de Souza Andrade performed the study of immunology. Elizângela Mírian Moreira performed the study of immunology. Renata Reis da Silva performed the study of immunology. Raquel Yvonne Arantes Baccarin participated in the development of stem cells. Lilian Rose Marques de Sá performed a histopathological analysis of the uterus. Lilian Gregory idealized and supervised the project.

## Funding

No funding was received for this manuscript.

## Disclosure

All authors read and approved the final manuscript.

## Ethics Statement

The Local Ethics Committee on the Use of Animals (CEUA) approved the project named “Evaluation of alternative treatments for the control of uterine infections in cattle” and all of the procedures performed in the experiment described in this manuscript (Protocol 2489230217) in the meeting of 10/18/2018.

## Consent

The authors have nothing to report.

## Conflicts of Interest

The authors declare no conflicts of interest.

## Data Availability

All data generated or analyzed during this study are included in this published article.
